# Intersphincteric Resection for Low Rectal Cancer: An Overview

**DOI:** 10.1155/2012/241512

**Published:** 2012-06-07

**Authors:** Constantine P. Spanos

**Affiliations:** 1st Department of Surgery, Aristotelian University School of Medicine, 55236 Thessaloniki, Greece

## Abstract

The treatment of rectal cancer has evolved from being solely a surgical endeavor to a multidisciplinary practice. Despite the improvement in outcomes conferred by the addition of chemoradiation therapy to rectal cancer treatment, advances in surgical technique have significantly increased rates of sphincter preservation and the avoidance of a permanent stoma. In recent years, intersphincteric resection for low rectal cancer has been offered and performed in patients as an alternative to abdominoperineal resection. An overview of this procedure, including indications, oncological and functional results based on current literature, is presented herein.

## 1. Introduction

There has been an evolution in the treatment of rectal cancer in recent times. A few decades ago, rectal cancer treatment was solely a surgical endeavour. Nowadays, it has evolved into therapy involving several disciplines. Nevertheless, surgery remains the cornerstone of curative treatment. The incorporation and widespread use of total mesorectal excision (TME) as the standard mode of surgical resection of adenocarcinoma of the rectum has been the most important surgical development in outcomes improvement for this disease [1, 2]. Supervised teaching of TME, as well as the detailed pathological audit of resected specimens, has also led to better oncological results [3]. Advances in surgical technique with the use of either advanced stapling or manual coloanal anastomoses have allowed for achieving continuity of the gastrointestinal tract at levels closer to the anal verge than those achieved historically. The advent of adjuvant and neoadjuvant chemoradiotherapy has also increased local control of disease [4] and in some instances has led to increased survival [5]. 

Surgery for rectal cancer in recent years has focused on anatomic and functional preservation of the sphincter without compromising oncological outcomes. Radical surgical treatment of cancers in lower third of the rectum has traditionally included low anterior resection (LAR) and coloanal anastomosis, and abdominoperineal resection (APR). Historically, the decision-making for sphincter-saving procedures has been related to the distance between the tumor and the anal sphincter complex [6]. In the 1980s, a distal margin of 5 cm was required. In the ensuing decades, the “2-cm-rule” was accepted and adopted [6]. This rule has been challenged, however, and currently a distal margin of 1 cm is accepted as being appropriate for optimal oncologic outcome. This provides a greater proportion of rectal cancer patients with the possibility of sphincter preservation [7, 8]. Recently, adequacy of the circumferential resection margin is being considered of equal, if not greater, importance in the risk of local recurrence of rectal cancer [9]. In recent years, intersphincteric resection (ISR) has been proposed to offer sphincter preservation in patients with very low rectal lesions, as an alternative to APR. Of note, APR has consistently had higher rates of local recurrence rates (up to 22%) compared with LAR [10, 11]. 

## 2. Materials and Methods

A literature search for relevant articles in the English language associated with intersphincteric resection (ISR) between 2000 and 2012 was undertaken. All articles regarding intersphincteric resection were caseseries from single institutions or systematic reviews. Case reports were excluded from this overview. Medline was the search engine utilized.

### 2.1. ISR: Definition

Schiessel and colleagues initially described the technique of ISR [12]. During ISR, a transanal division of the rectum, with removal of part or the entire internal anal sphincter (IAS) after TME, is performed, thus obtaining an adequate distal margin. Restoration of bowel continuity is achieved by performing a hand-sewn coloanal anastomosis.

## 3. Indications and Preoperative Evaluation

When planning for proctectomy with ISR for rectal cancer, careful patient selection is paramount. Tumor height, its relationship to each component of the sphincter complex, and the presence or not of regional lymph node or distal metastases needs to be evaluated. For this reason, a combination of a careful physical exam and imaging modalities is utilized. Preoperative evaluation by the surgeon by means of digital rectal exam and rigid proctoscopy provides information regarding the level of the distal edge of tumour relative to the “anal anatomic component of interest,” which varies among experts in the literature [13, 14]. Anal anatomic components of interest include the anal verge, the dentate line and the anorectal ring. Specialized imaging is required to study the relationship of the IAS and external anal sphincter (EAS) with the tumour. Invasion of these structures by the lesion can also be depicted. Endorectal/endoanal ultrasound and magnetic resonance imaging (MRI) are performed for this reason. In addition, high-resolution MRI is accurate at estimating the circumferential margin; with an overall accuracy of 88% [15]. Additional cross-sectional imaging evaluates the presence of distal metastases.

Inclusion criteria for performance of ISR include the following:

- tumours located 30 mm from anal verge;
- tumours located 15 mm from dentate line;
- tumours located 1 cm from anorectal ring;
- local spread restricted to the rectal wall or the IAS;
- adequate preoperative sphincter function and continence;
- absence of distant metastases.

Contraindications to the performance of ISR are the presence of fecal incontinence, T4 lesions, undifferentiated tumors [10], as well as tumors invading the puborectalis and the external anal sphincter (EAS) [14]. 

A significant number of patients may require neoadjuvant chemoradiation therapy. In a systematic review of ISR involving 14 studies and 1289 patients who underwent ISR by Martin and colleagues, 44% of patients had stage III disease and 38% underwent preoperative chemoradiation overall [14]. Of note, in certain studies included in the review, preoperative radiation was a contraindication to performing ISR due to possible adverse functional effects. This is in contrast to a recent study by Denost and colleagues in which 93% of patients undergoing ISR received preoperative radiotherapy [16]. 

### 3.1. Surgical Technique

The principle of the ISR technique is based on an anatomic dissection plane between the IAS and EAS [17]. 

The technique incorporates a combined abdominal and perineal approach. Initially, high ligation of the inferior mesenteric vessels is done. This is followed by TME down to the level of the pelvic floor. TME can be performed through a laparotomy or laparoscopically [6]. Subsequently, a per anal resection of the IAS is undertaken. The distal resection line may be at the intersphincteric groove (total ISR), between the dentate line and the intersphincteric groove (subtotal ISR), or at the dentate line (partial ISR).

Additional maneuvers to reduce the risk of local tumor cell implantation include closure of the rectal stump, cytocidal washout, and pathological evaluation of the distal margin with frozen section analysis [18]. 

The specimen is usually delivered per anum. A hand-sewn coloanal anastomosis with construction of a colon J-pouch, transverse coloplasty, or straight anastomosis is performed.

Certain groups, especially in Japan, perform lateral pelvic lymph node dissection for TNM stage III tumors [13]. 

A defunctioning temporary stoma is fashioned, which is closed 6 weeks to 12 months from the primary operation.

### 3.2. Short-Term Adverse Events

The overall operative mortality associated with ISR is 0.8% [14]. The cumulative morbidity rate is reported to be 25.8%. Anastomotic leak was experienced after a mean of 9.1%, and the rate of pelvic sepsis was 2.4% [14]. 

The rate of clinically apparent anastomotic leakage following stapled anastomosis following anterior resection is in the range of 3–15%. Rates of leakage rise significantly for more distally sited anastomoses [19]. Anastomotic leakage is associated with postoperative anastomotic stricture, cancer recurrence, poor postoperative function, as well as increased operative mortality [20]. 

In conclusion, ISR can be performed with acceptable rates of anastomotic leakage and low operative mortality. 

### 3.3. Oncologic Outcomes

Radical surgical removal of the tumor is the only chance for permanent cure of rectal cancer, despite all progress in the development of oncologic therapy [10]. Rullier and colleagues [6] reported a local recurrence rate of 2% in a series of 92 patients undergoing ISR. Most patients (78%) had T3 lesions, and 88% underwent long-course neoadjuvant radiochemotherapy. The overall 5-year survival rate was 81%, with a 5-year disease-free survival of 70%. Yamada et al. reported a similarly low 2.5% cumulative 5-year local recurrence rate, a 5-year disease-free survival rate of 83.5% for stage II patients and 72% for stage III patients [13]. 

Tilney and Tekkis performed a literature search to identify studies reporting outcomes following ISR. Twenty-one studies accumulating a total of 612 patients were identified [20]. The pooled rate of local recurrence was 9.5% with an average 5-year survival of 81.5%. Distant metastases occurred in 9.3%. In Martin's systematic review, the mean distal margin free from tumour was 17.1 mm, CRM-negative margins were achieved in 96% of patients, and the overall local recurrence rate was 6.7% (range: 0–23%). The 5-year overall and disease-free survival rate was 86.3% and 78.6%, respectively [14]. 

Rates of local recurrence following low anterior resection for the treatment of rectal cancer are commonly reported in the range of 2.6–32% following surgery alone [21]. Preoperative chemoradiation therapy has led to local recurrence rates in the 6% range [4]. 

Therefore, the performance of ISR for treatment of very low rectal cancer affords similar oncologic outcomes to those of conventional resections. Moreover, Saito et al. [22] compared outcomes of patients undergoing ISR with patients undergoing APR. Similar local recurrence rates (ISR = 10.6%, APR = 15.7%, *P* = NS) and 5-year disease-free survival (ISR = 69.1%, APR = 63.3%, *P* = NS) were reported. Patients undergoing ISR had significantly longer 5-year overall survival compared with patients undergoing APR (ISR = 80%, APR = 61.5%, *P* < 0.05). In conclusion, local and distant oncologic outcomes are not comprised with ISR. It is considered that the risk of local recurrence may be a function of circumferential margin involvement rather than distal margin involvement.

Risk factors for local and distant recurrence after ISR were reported by Akasu et al. [23]. Local recurrence rate was 6.7% and distant recurrence was 13%.

In the multivariate analysis, risk factors for local recurrence included positive microscopic resection margins, focal d-differentiation of tumor (tumor budding), and elevated preoperative levels of CA 19-9 (>37 U/mL). The identified risk factors for distant recurrence were pN1, pN2 disease, poor differentiation, and distance of tumor from anal verge, 2.5 cm.

### 3.4. Anorectal Physiology

An important goal of sphincter-preserving surgery is to reach acceptable quality of life levels by preserving fecal continence. The main concern of the ISR technique is functional outcome. Physiologic studies have shown that anal resting pressure is due to the IAS for 55%, the hemorrhoidal plexus for 15%, and to the EAS for 30% [24]. 

Total or partial excision of the IAS is bound to affect continence. Furthermore, preoperative radiation therapy may cause additional loss of sphincter function.

Kohler et al. reported a 29% reduction in resting anal pressure following ISR. Squeeze pressure recovered to preoperative levels after 12 months [25]. 

Rullier et al. compared outcomes in patients undergoing partial or subtotal IAS resection. Subtotal excision of the sphincter was associated with significant reduction in resting but not squeeze pressure after ISR [26]. Of note, there have been no studies assessing anorectal physiology and continence after neoadjuvant radiation and prior to ISR.

### 3.5. Functional Outcomes and Quality of Life

As an antithesis to an aphorism by the famed architect Louis Sullivan, in rectal cancer surgery, “function follows form” (the type of operation performed). Loss of a part of the sphincter complex, loss of the rectal reservoir, and radiation is bound to have adverse effect on continence and defractory function.

Bretagnol and colleagues reported that fecal continence measured by both the Kirwan and Wexner scores was significantly worse after ISR. In addition, the need for antidiarrheal medication was higher in patients undergoing ISR compared with patients that had undergone conventional coloanal anastomosis [27]. 

Frequency, urgency, the Wexner score, and the Fecal Incontinence Severity Index (FISI) were significantly improved following colonic J-pouch reconstruction compared with straight coloanal anastomosis [27]. 

Regarding quality of life (QOL), Bretagnol et al. used both the SF-36 and fecal incontinence quality of life (FIQL) to compare QOL between patients undergoing ISR and conventional coloanal anastomosis. There was no difference in the QOL scores between ISR patients and conventional coloanal anastomosis patients in the physical and mental subscales of the SF-36.

In Martin's systematic review, the mean number of bowel movements per day was 2.7. Nearly half (51.2%) of patients reported “perfect continence,” about a third (29.1%) reported experienced fecal soiling, 23.8% had flatus incontinence, had 18.6% had urgency. In a large study assessing functional outcomes after ISR, Denost reported that half of the patients had a “good functional result,” 39% had minor fecal incontinence, and 11% had major incontinence [16]. In the same study, the only independent predictors of “good” continence were a distance of tumour greater than 1 cm from the anorectal ring and anastomosis higher than 2 cm from the anal verge.

Possible technical modifications when performing ISR may improve functional outcomes. These include partial ISR (when possible) and construction of a colon J-pouch. These are known to improve function in the first year after surgery. However, the effect is not sustained after 2 years [14]. 

## 4. Conclusion

In order to be successful in treating rectal cancer, good oncologic outcome is the first priority. Equally important is the achievement of an acceptable quality of life for the patient. The avoidance of a permanent stoma and all of the concomitant morbidity associated with it may be of greater importance to the patient. Low anterior resection with intersphincteric dissection and partial or total excision of the IAS may be offered an alternative to APR in selected patients. The functional outcomes after ISR are expected to be inferior to those of conventional low anterior resection; this information needs to be frankly communicated to the patient. The morbidity, mortality, and oncological outcomes after ISR are acceptable. Careful patient selection and sound operative technique, with emphasis on high-quality preoperative imaging and functional assessment, should lead to superior results. These principles have been closely examined at our own institution, and we have embarked on our first cases of intersphincteric resection in selected patients.
